# New journal: Algorithms for Molecular Biology

**DOI:** 10.1186/1748-7188-1-1

**Published:** 2006-02-28

**Authors:** Burkhard Morgenstern, Peter F Stadler

**Affiliations:** 1 Universität Göttingen, Institut für Mikrobiologie und Genetik, Abteilung für Bioinformatik, Goldschmidtstrasse 1, D-37077 Göttingen, Germany; 2 Universität Leipzig, Institut für Informatik und Interdisziplinäares Zentrum für Bioinformatik, Kreuzstrasse 7b, D-04103 Leipzig, Germany

## Abstract

This editorial announces *Algorithms for Molecular Biology*, a new online open access journal published by BioMed Central. By launching the first open access journal on algorithmic bioinformatics, we provide a forum for fast publication of high-quality research articles in this rapidly evolving field. Our journal will publish thoroughly peer-reviewed papers without length limitations covering all aspects of algorithmic data analysis in computatioal biology. Publications in *Algorithms for Molecular Biology *are easy to find, highly visible and tracked by organisations such as PubMed. An established online submission system makes a fast reviewing procedure possible and enables us to publish accepted papers without delay. All articles published in our journal are permanently archived by PubMed Central and other scientific archives. We are looking forward to receiving your contributions.

## 

This editorial is the first article published in our new journal *Algorithms for Molecular Biology*. By starting this journal, we aim to provide an online and open access resource for the growing research community in the field of algorithmic bioinformatics. Bioinformatics or computational biology is a very broad and heterogeneous field of research ranging from applied data analysis and IT support for life-science projects to probabilistic modelling, algorithm development and complexity analysis. Today, there is a variety of established and newly founded bioinformatics journals covering these diverse areas. Some of these journals are general-purpose journals covering the whole range of research topics in computational biology, for example *Bioinformatics *or *BMC Bioinformatics*. Other journals are specialised on applied bioinformatics where software tools are used as a means to obtain biological insights, e.g. *In Silico Biology *and *PLoS Computational Biology*. There are also some existing journals that focus on algorithmic topics in bioinformatics, e.g. *Journal of Computational Biology*, *Journal of Bioinformatics and Computational Biology *or *IEEE/ACM Transactions on Computational Biology and Bioinformatics*. These algorithmic journals are run in the traditional way where publishing is free of charge but readers or their libraries have to pay subscription fees to obtain access to published research results.

During the last few years, online open access journals have become popular in many areas of research. In contrast to established publishing models, these journals provide free and unlimited access to research articles for everyone connected to the internet. Online publishing offers a rapid way of publishing research results since every article is ready to be published immediately after formal acceptance. Above all, articles in open access journals are highly visible since access is not limited to those whose libraries can afford increasingly expensive subscription fees. Publication costs for open access journals are defrayed by authors or their institutions, either by an article processing charge per published article, or by membership fees where institutions pay a fixed amount per year covering all articles published by their employees. In all life sciences there are now journals adopting an open access publishing policy, and it is apparent that, after some initial scepticism, a growing number of scientists now realise the benefits of this new publishing model. Open access publishing is also supported by public funding agencies in an effort to limit ever increasing subscription fees. As for traditional print journals, publications in online journals are tracked by services such as *PubMed *or *Google Scholar *and many of them already have good impact factors or are on their way to receiving one.

While open access journals are becoming successful in all areas of science, the success of this new publishing model is particularly striking in bioinformatics. Here, there is a strong tradition of open-source software, and most academic research groups make their data and software freely available through the internet. We think that in some way, open source and open access belong together; this may be one reason for the rapid success of open access publishing in bioinformatics. The idea is that the results of publicly funded research efforts should be freely available to the general public. A good example is the journal *BMC Bioinformatics *which was established in 2000 [2]. During the first few years, only a small number of articles were published in this journal, but the 2005 volume already contains as many as 311 regular articles plus four supplements, and in January 2006 alone, 49 articles were published in the journal. In 2005, *BMC Bioinformatics *received its first official impact factor which was an impressive 5.42 comparable to the established journal *Bioinformatics *which has been in existence for more than 20 years and is one of the leading journals in the field [3].

*Algorithms for Molecular Biology *is the first online open access journal in the field of algorithmic bioinformatics. Our journal will publish thoroughly peer-reviewed articles of high scientific quality on novel algorithms and software tools for molecular biology, genomics and proteomics. Areas of interest include but are not limited to: RNA and protein structure prediction and analysis, gene prediction and genome analysis, machine learning, combinatorial algorithms, comparative sequence analysis and alignment, phylogeny and gene expression. The journal has currently two *Editor-in-Chiefs *and six *Associate Editors*, each of whom is responsible for one of the above research areas. In addition, the journal has an Editorial Board consisting of internationally renowned experts in the field.

Most publications in our journal will be scientific articles. But from time to time, we will also publish reports, commentaries etc. on topics of general interest to the readership of the journal. Currently, we have the following article categories:

• *Research articles: *these articles should present original research work on the development and analysis of novel algorithms in bioinformatics. In general, the usefulness of algorithms should be demonstrated by applications to biological data. However, purely theoretical manuscripts are also welcome if future applications to problems in molecular biology are to be expected or if they address complexity or approximation issues of novel computational problems in molecular biology. Clear writing and understandable algorithm description are of particular importance. Unclearly written manuscripts will be returned to the authors regardless of their scientific content.

• *Software articles: *these papers should describe newly developed software tools or substantial improvements to existing tools. They should contain some algorithmic novelty; manuscripts about web servers, graphical user interfaces etc. will not be considered.

• *Short reports: *brief reports of original research work.

• *Editorials: *short articles by members of the editorial team about general questions concerning the journal.

• *Review articles: *these articles should give a general overview on a sub-field of algorithmic bioinformatics. In general, reviews are invited by the Editorial Board, but proposals for review articles are welcome.

• *Book reports: *short summaries of the strengths and weaknesses of a book; they should evaluate its overall usefulness to the intended audience. As review articles, book reports are usually invited by the editors.

• *Commentaries: *short, focused and opinionated articles on any subject within the journal's scope. These articles are usually related to a contemporary issue, such as recent research findings, and are often written by opinion leaders invited by the Editorial Board.

• *Debate articles: *these articles present an argument that is not essentially based on practical research. Debate articles can report on all aspects of the subject including political, sociological and ethical aspects.

• *Meeting reports: *short reports on conferences that the authors have attended, usualy invited by the Editorial Board. It is best for these articles to be published as soon after the meeting as possible, and they should focus on the key developments presented and discussed at the meeting.

There are many reasons to publish in our new journal. All articles in *Algorithms for Molecular Biology *are fully open access according to the BioMed Central Charter [1], so they are universally accessible online without charge. An electronic submission and peer-review system guarantees a rapid and efficient reviewing process, and there are no length limitations for text, figures and additional material. Manuscripts accepted for publication are published without delay, and all published articles are permanently archived in PubMed Central, the free-access repository of peer-reviewed research in the life sciences run by the National Institutes of Health, USA, as well as other national archives. This way, articles in our journal are highly visible and easy to find, read and cite – an important advantage in times where citation rates are becoming increasingly important for funding and promotion in science. *Algorithms for Molecular Biology *is looking forward to receiving your submissions.

## Competing interests

We do not have any competing interests. In particular, we want to emphasise that neither we nor any member of our editorial team nor our institutions will have any financial gains from running this journal.

## References

[B1] BioMed Central Open Access Charter. http://www.biomedcentral.com/info/about/charter.

[B2] Cockerill MJ (2005). PBMC Bioinformatics comes of age. BMC Bioinformatics.

[B3] Valencia A, Bateman A (2005). Increasing the impact of Bioinformatics. Bioinformatics.

